# Ultrathin Ceramic Membranes as Scaffolds for Functional Cell Coculture Models on a Biomimetic Scale

**DOI:** 10.1089/biores.2015.0037

**Published:** 2015-12-01

**Authors:** Corinne Jud, Sher Ahmed, Loretta Müller, Calum Kinnear, Dimitri Vanhecke, Yuki Umehara, Sabine Frey, Martha Liley, Silvia Angeloni, Alke Petri-Fink, Barbara Rothen-Rutishauser

**Affiliations:** ^1^ Adolphe Merkle Institute, University of Fribourg, Fribourg, Switzerland.; ^2^ Agroscope, Institute for Livestock Sciences ILS, Posieux, Switzerland.; ^3^ CSEM SA, Neuchâtel, Switzerland.; ^4^ University Children's Hospital Basel, Basel, Switzerland.

**Keywords:** cell culture, tissue engineering, toxicology

## Abstract

Epithelial tissue serves as an interface between biological compartments. Many *in vitro* epithelial cell models have been developed as an alternative to animal experiments to answer a range of research questions. These *in vitro* models are grown on permeable two-chamber systems; however, commercially available, polymer-based cell culture inserts are around 10 μm thick. Since the basement membrane found in biological systems is usually less than 1 μm thick, the 10-fold thickness of cell culture inserts is a major limitation in the establishment of realistic models. In this work, an alternative insert, accommodating an ultrathin ceramic membrane with a thickness of only 500 nm (i.e., the Silicon nitride Microporous Permeable Insert [SIMPLI]-well), was produced and used to refine an established human alveolar barrier coculture model by both replacing the conventional inserts with the SIMPLI-well and completing it with endothelial cells. The structural–functional relationship of the model was evaluated, including the translocation of gold nanoparticles across the barrier, revealing a higher translocation if compared to corresponding polyethylene terephthalate (PET) membranes. This study demonstrates the power of the SIMPLI-well system as a scaffold for epithelial tissue cell models on a truly biomimetic scale, allowing construction of more functionally accurate models of human biological barriers.

## Introduction

In the field of regulatory toxicology, animal testing is the standard approach to test possible adverse effects of chemicals or drugs.^1^ New concepts for more efficient, cheaper, and evidence-based test strategies have been proposed, such as a shift from phenomenological analyses in animals toward mechanism-based assays using human primary cells and cell lines.^2^ The lung is the main portal of entry for inhaled aerosols^3^ and is therefore a promising pathway for the inhalation of drugs.^4^ Attention has recently been directed toward elucidating how aerosol-based pharmaceuticals interact with the lung barrier, many cell models having been established to address this question.^5^

*In vitro* cocultures mimicking the alveolar–capillary barrier with two cell types, that is, epithelial and endothelial cells (either primary cells or cell lines), have been described previously.^6–8^ Another development focused on the design of a “lung-on-a-chip” setup to reconstitute the alveolar–capillary interface of the human lung with cocultures under flow and breathing conditions, that is, mechanical stress.^9,10^ In addition to the barrier structure, other models have started to include immune cells to mimic the innate and adapted immune response to the inhalation of xenobiotics, such as macrophages and dendritic cells,^11^ macrophages and mast cells,^12,13^ or natural killer cells.^14^ The previously described cocultures of the air–blood tissue barrier represent well-defined and physiologically relevant *in vitro* models.

However, these models all have one common limitation: a several-micron-thick microporous membrane as a support for the cells to grow on. Given that the air–blood barrier in humans has a mean arithmetic thickness of 2.2 μm and can span less than 1 μm,^15,16^ these thick mechanical supports almost certainly influence cell–cell interactions very strongly, as well as the translocation characteristics of any particle or drug that is deposited on the apical surface of the cell cultures, for three main reasons. First, from a biological point of view, the overall barrier architecture is affected and thus presumably also its structural–functional behavior. Second, from a physical point of view, the time taken for any xenobiotic (e.g., a drug/an aerosol) to diffuse over a certain distance increases with the square of the distance, leading in at best to a nonnegligible impact on the translocation kinetics.^17–19^ Third, the large internal surfaces of the membrane may adsorb xenobiotics, blocking the micropores and preventing translocation of any species.

The aims of this work were to design a thin, optically transparent, and mechanically robust permeable membrane and to demonstrate its potential in a functioning alveolar–capillary barrier cell culture system. A permeable support consisting of a silicon network framing an array of 23 silicon nitride (ceramic) freestanding microporous membranes were microfabricated, each having a thickness of 500 nm.^18^ The resulting Silicon nitride Microporous Permeable Insert (SIMPLI-well) system has been patented by the CSEM SA.^20^ Furthermore, the ceramic chip can be easily flipped, facilitating the culturing of different cell types on opposite sides of the membrane. Quadruple cultures composed of epithelial–endothelial bilayers supplemented with two immune cells, macrophages and dendritic cells, were optimized and characterized with regard to cell growth, morphology, and membrane integrity. In addition, and to validate the system, the translocation behavior of polyvinyl alcohol (PVA)-coated gold nanoparticles (AuNPs) with a hydrodynamic diameter of 42.2 nm was investigated in quadruple cocultures grown on either commercially available polyethylene terephthalate (PET) membranes or SIMPLI-wells.

## Materials and Methods

### Design and fabrication of the SIMPLI-well system

The SIMPLI-well holder was micromachined according to a design proprietary to the CSEM^21^ in polycarbonate (PC; 1000 Angst+Pfister AG) and was successfully tested for sterilization by autoclaving through extensive cleaning with isopropanol and water as issued from fabrication (i.e., residual handling and machine oil). The porous supports for cell culture were fabricated using a standard microfabrication process as described previously.^22^

Briefly, 500 nm of low-stress (nonstoichiometric) silicon nitride (SixNy) is deposited on both sides of a 380-μm-thick silicon wafer by low-pressure chemical vapor deposition. Photolithography defines structures on both sides of the wafer that are etched into the silicon nitride by reactive-ion etching. The structures on the top side define the pore size, shape, and period in the porous support. These features were inspected by scanning electron microscopy (SEM XL-40 Philips). In this specific chip layout, on the other side of the wafer, square openings of 1.5 × 1.5 mm^2^ in the silicon nitride are used as a mask for a wet KOH etch that removes the exposed silicon and releases the porous silicon nitride supports as microporous membranes of size 1 × 1 mm^2^ upon going through the pyramidal anisotropic etching. Individual 14 × 14 mm^2^ chips were obtained upon dicing. To remove microfabrication process residues, the chips were cleaned in a hot Piranha solution (98% H_2_SO_4_ and 30% H_2_O_2_ in a ratio of 4:1) at 110°C, followed by extensive rinsing with deionized water and drying under laminar flow (Please note that the Piranha solution is a strong oxidizing substance and must be prepared by care. Consult the Laboratory Safety Coordinator before the solution is prepared.). The array of porous silicon nitride windows is mechanically supported by the surrounding silicon chip. We will refer to the whole as silicon nitride porous supports or ceramic chips or ceramic substrates, emphasizing the silicon nitride interface, which is in contact with the cell lines. The SIMPLI-well fits in a standard six-well cell culture plate.

### Pretreatment and regeneration of the SIMPLI-well

Before the cell culture experiments, the silicon nitride porous supports were subjected to a standard clean 1 (SC-1). The membrane chips were placed on a Teflon holder and incubated for 10 min in a 70°C mixture of Milli-Q water, HN_4_OH (28%), and H_2_O_2_ (30%) at a ratio of 4:1:1. The strong oxidizing potential of this solution ensures that the chip surface is free from organic (as well as some metallic) contaminants. After the SC-1 treatment, the chips were washed extensively with Milli-Q water. After completion of the cell experiments, the porous supports were cleaned, repeating the steps described above starting with a Piranha treatment.

The PC moieties of the SIMPLI-wells were placed in an ultrasound bath for 15 min in Milli-Q water, 15 min in isopropanol, and another 1 min in Milli-Q water. Membrane chips and PC moieties that were exposed to AuNPs were additionally washed three times for 2 min with 5 mM KCN and rinsed extensively with Milli-Q water before reuse.

### Cell cultures

**Note:** Where not specified, the same protocols were used for both PET inserts and SIMPLI-wells.

Experiments were performed with the human alveolar epithelial type II cell line A549^23^ (American Type Culture Collection) and the endothelial cell line EA.hy926, which was obtained by fusion of human umbilical vein cells with a thioguanine-resistant clone of A549^24^ (provided by Dr. Edgell, University of North Carolina). A549 cells were cultured in RPMI 1640 containing HEPES (GIBCO; Invitrogen) supplemented with 10% heat-inactivated fetal bovine serum (FBS Gold; PAA Laboratories), 1% l-glutamine (GIBCO; Invitrogen), and 1% penicillin/streptomycin (GIBCO; Invitrogen) and maintained at 37°C and 5% CO_2_. Cells were split twice a week with trypsin (0.05% trypsin–EDTA; GIBCO; Invitrogen) and seeded 1:16 in 75-cm^2^ cell culture bottles (TPP; Milian). EA.hy926 cells were cultured in DMEM containing high glucose, sodium pyruvate, and l-glutamine (GIBCO; Invitrogen) supplemented with 10% FBS and 1% penicillin/streptomycin. Cells were maintained at 37°C and 5% CO_2_ and were split twice a week with trypsin and seeded 1:8 in 75-cm^2^ cell culture bottles.

Peripheral blood monocytes were isolated from buffy coats (Blood Donation Service SRK, Bern AG, Switzerland) using Lymphoprep™ density gradients and CD14+ MicroBeads (Miltenyi Biotec GmbH) according to the manufacturer's manual. For the generation of monocyte-derived dendritic cells (MDDCs), the monocytes were cultured for 7 days in RPMI complete media with additional supplementation of 10 ng/mL IL-4 (R&D Systems Europe Ltd.) and 10 ng/mL GM-CSF (R&D Systems Europe Ltd.). Monocyte-derived macrophages (MDMs) were obtained by culturing the monocytes for 7 days in RPMI complete media containing 10 ng/mL M-CSF (R&D Systems Europe Ltd.).

### Co- and quadruple cultures

#### PET membranes

Conventional 12-well cell culture inserts (PET, pore size: 1 or 3 μm; BD Falcon; Milian) were turned upside down and placed in sterile Petri dishes before 0.5 × 10^6^ EA.hy926 cells per 0.9 cm^2^ were seeded on the basal side of the PET membranes. Cells were allowed to adhere for 90 min in the incubator. After removing nonadherent cells, 12-well inserts were placed in 12-well plates (BD Falcon; Milian), and then, 2 mL DMEM was added to the lower chamber and 1 mL to the upper chamber. EA.hy926 cells were cultured for 1 day, and then, all medium of the 12-well plates was removed and fresh DMEM was added to the lower chamber before 0.5 × 10^6^ A549 cells per 0.9 cm^2^ were seeded to the upper chamber, and the volume was filled up to 1.5 mL with RPMI medium. The medium was changed every second day while double cocultures were allowed to stabilize. On day 8, MDDCs were added to the basal, and MDMs to the apical, sides of each membrane. For this, the medium was removed, and the inserts were turned upside down and placed in sterile Petri dishes. MDDCs were harvested, and 60,000 cells were added in a cell suspension not exceeding 200 μL to the basal side of each membrane. Cells were allowed to attach for 60 min. Then, excess medium was removed, and the inserts were placed into new culture plates. A mixture of 70% DMEM and 30% RPMI was used to culture the cells, and 2 mL was added to the lower chamber. A total of 12,000 MDMs were added to the upper chamber of each insert, and the volume was filled up to 1.5 mL with the medium mixture. The quadruple cocultures were incubated for 24 h at 37°C and 5% CO_2_.

#### SIMPLI-wells

The cocultures have been assembled similarly to those on conventional PET membranes with some exceptions: SIMPLI-wells containing SC-1-cleaned CSEM membrane chips (mounted flat side up) were autoclaved and incubated for 1 day in supplemented DMEM cell culture medium (six-well plate, 4.5 mL bottom and 1.5 mL top). 0.5 × 10^6^ EA.hy926 cells per 0.8 cm^2^ were seeded. After 1 day of growth, the SIMPLI-well was disabled, and the ceramic chip hosting the first adherent layer of endothelial cells was kept in prewarmed DMEM. Then, the PC clamping system was dipped in water for a few minutes, sterilized in 70% ethanol, and washed. The ceramic chips were then reclamped, thanks to PC moieties sliding one into the other, assuring that the SIMPLI-wells are remounted the other way around with the flat side (covered with EA.hy926 cells) now facing down. Complete 4.5 mL DMEM was added to the bottom of each SIMPLI-well before 0.5 × 10^6^ A549 cells were seeded on the multiwell side (380.5 μm deep) of the silicon nitride chip (upper chamber). The volume of the upper chamber was filled to 1.5 mL with RPMI medium. The addition of MDM and MDDC was performed similarly to that described for the PET membranes.

### Lactate dehydrogenase assay

To determine cytotoxicity, the supernatant was sampled and stored at 4°C for the lactate dehydrogenase (LDH) assay. Triton X-100 detergent (0.2% in medium) was used for cell lysis as a positive control. The supernatant of untreated cells was used as a negative control. The LDH assay was performed with the Cytotoxicity Detection Kit (Roche Applied Science) according to the supplier's manual. Samples were diluted 1:10. LDH was quantified photometrically by measuring at 490 nm, with 630 nm as the reference wavelength. Each sample was assessed in triplicate. The values were expressed as a fold increase related to the incubator control at appropriate postexposure times.

### Dextran blue assay

Blue Dextran 2000 (GE Healthcare; about 2000 kDa) was used to assess membrane integrity and tight junction formation of the co- and quadruple cultures as described elsewhere.^25^ The cell culture medium was removed, and the cells were washed once with 1× phosphate-buffered saline (PBS; GIBCO; Invitrogen). Then, 0.5 mL supplemented phenol red-free medium was added to the upper, and 1 mL to the lower, chamber. To each upper chamber, 0.5 mL of 1% Blue Dextran 2000 in PBS was added, and the cells were incubated for 2 h at 37°C and 5% CO_2_. The content of each lower chamber was collected, and the optical densities were determined photometrically (600 nm). As a reference value, insert-only controls (with no cells) were used. Cultures treated with 2 mM EDTA for 2 h were used as controls as described earlier.^25^ Supplemented phenol red-free medium was used as a blank.

### Fluorescent microscopy

**FIG. 1.**
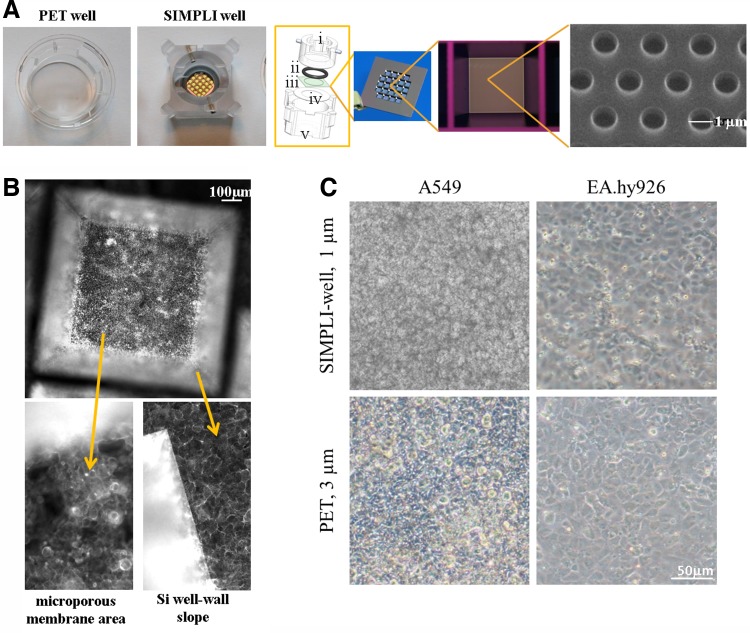
Characterization of the silicon nitride porous supports and cell growth. **(A)** From left to right: polyethylene terephthalate (PET) well; Silicon nitride Microporous Permeable Insert (SIMPLI)-well; schematic view of an SIMPLI-well composed of upper polycarbonate cylinder (i), silicon O-ring (ii), ceramic chip (iii), Teflon antiwear joint (iv), and lower cylindrical gasket (v)—the two cylindrical moieties slide one into the other and tightly clamp the ceramic chip through a bayonet locking system; ceramic chip of dimensions 14 mm × 14 mm displaying an array of 23 permeable wells; 1 mm × 1 mm permeable well; scanning electron microscopy image of adjacent pores with a diameter of 1 μm in hexagonal pattern, yielding a 15% filling factor. **(B)** Fluorescence pictures of A549 epithelial cells grown for 7 days on the silicon nitride porous support showing the growth of cells on different areas, such as the silicon (Si) well slope and the silicon nitride flat membrane in the permeable well bottom (1 × 1 mm^2^). The F-actin cytoskeleton has been stained with rhodamine phalloidin (shown in white). **(C)** Phase-contrast images of A549 epithelial and EA.hy926 endothelial cells grown on the silicon nitride porous supports and conventional PET membranes with 3-μm pores.

A Nikon fluorescence microscope with CCD camera (F-View II FireWire™ fluorescence camera) and FIVE software (Olympus Schweiz AG) was used for the images in Figure 1B.

### Laser scanning microscopy

For laser scanning microscopy (LSM) analysis, insert membranes containing the cells were fixed with 3% paraformaldehyde (PFA, Sigma-Aldrich) in PBS for 15 min at room temperature. Then, cells were incubated in 0.1 M glycine in PBS for 40 min, washed with PBS for 5 min, and further permeabilized for 15 min with 0.2% Triton X-100 in PBS. After a further washing step with PBS, the primary antibodies were applied overnight at 4°C at a concentration of 1:100 in 0.1% Triton X-100 and 1% bovine serum albumin (BSA) in PBS:polyclonal rabbit anti-human Von Willebrand factor (vWF, H-300, sc-14014; Santa Cruz Biotechnology) and monoclonal mouse anti-human platelet endothelial cell adhesion molecule-1 (PECAM-1, 10G9, sc-13537; Santa Cruz Biotechnology). Membranes were rinsed three times with PBS before the secondary antibody, cytoskeleton and DNA staining was applied at room temperature in the dark for 3 h at the following concentrations in 0.1% Triton X-100 and 1% BSA in PBS:polyclonal goat anti-rabbit cyanine-5 1:50 (Chemicon, VWR International AG, Life Sciences), polyclonal goat anti-rabbit DyLight649 1:50 (Merck Millipore), polyclonal goat anti-mouse cyanine-2 1:50 (Chemicon, VWR International AG, Life Sciences), rhodamine phalloidin 1:100 (Molecular Probes; Invitrogen), and DAPI at 1 μg/mL (Molecular Probes; Invitrogen). Afterward, the cells were washed twice with PBS and once with Milli-Q water and mounted on glass microscope slides in Glycergel mounting medium (DakoCytomation). Silicon nitride porous supports were mounted between two cover-slips. Analysis was performed with an inverted Zeiss LSM 510 Meta (Axiovert 200M, Zeiss) equipped with Argon/2 488 nm, HeNe 543 nm, and HeNe 633 nm lasers.

### Transmission electron microscopy

The cells were fixed with 2.5% glutaraldehyde in 0.15 M HEPES buffer (pH = 7.4) for at least 24 h, washed with HEPES buffer, postfixed with 1% osmium tetroxide in sodium cacodylate buffer, washed with maleate buffer, and stained en bloc with 0.5% uranyl acetate in maleate buffer. Afterward, the cells were dehydrated in ascending ethanol series and embedded in Epon. From the embedded cells, ultrathin sections were cut parallel to the vertical axis of the cells, mounted on copper grids, and stained with lead citrate and uranyl acetate. Imaging was done with a Morgagni TEM (FEI, Hillsboro, OR, USA) at 80 KV equipped with a Morada digital camera (Olympus SIS, Tokyo, Japan).

### Synthesis and characterization of AuNPs

All glassware was cleaned with aqua regia and extensively rinsed with ultrapure water before use. AuNPs (radius core: 7.8 nm, shell: 13.3 nm, and number-weighted polydispersity: 31.5%) were synthesized by a citrate reduction method.^26^ In brief, a solution of sodium citrate (50 mL, 38.8 mM) was added rapidly with magnetic agitation to a boiling solution of HAuCl_4_·3H_2_O (500 mL, 1 mM). Heating was continued for 15 min to ensure the complete reduction of all ionic gold. These citrate-coated nanoparticles were then coated with terminal thiol-functionalized PVA (M205; Kuraray Europe GmbH) by mixing the suspension with an aqueous solution of PVA at a concentration of 10 molecules/nm^2^ of NP surface area. The functionalized nanoparticles were suspended in 1× PBS (GIBCO; Invitrogen) at a stock concentration of 20.2 nM. Before use, the dispersions were placed in an ultrasound bath for 5 min and filtered through a 0.2-μm polyethersulfone (PES) filter (Acrodisc syringe filters with Supor membrane, 13 mm; Pall).

Particle core size distribution was obtained by image analysis of transmission electron microscopy (TEM) images using Fiji ImageJ. The hydrodynamic radius was assessed by depolarized dynamic light scattering (DDLS) using a 3D LS Spectrometer equipped with a polarizer situated in front of the detector (LS Instruments AG). Optical characterization was carried out by UV–Vis spectroscopy on a Jasco V-670 spectrophotometer. The UV–Vis spectra were acquired in water and PBS 1× to assess the colloidal stability. The surface charge of citrate- and polymer-coated AuNPs was measured in 10 mM PBS (pH 7) and water (pH 6) at 25°C using a phase amplitude light scattering (PALS) zeta potential analyzer (Brookhaven ZetaPALS) (see Supplementary Fig. S1).

### Cell exposure to AuNPs

The medium was removed from the quadruple cultures, and a mixture of phenol red-free 70% DMEM and 30% RPMI was prepared. Two milliliters of this mixture was added to the bottom of the SIMPLI-well and 0.9 mL to the bottom of the conventional 12-well inserts. One milliliter of AuNP suspension in phenol red-free medium mix at a concentration of 22.3 μg/mL was added to the top of each insert, and the cells were incubated with this suspension for 2 h at 37°C and 5% CO_2_. Medium mixed with PBS was used for control experiments. After incubation, the lower and upper chamber contents were harvested. In the upper chamber, the cells were washed three times with 500 μL PBS. The washing solution was kept for further analysis.

### Particle translocation

AuNP translocation was assessed by tracing the metal nanoparticle core using inductively coupled plasma optical emission spectroscopy (ICP-OES) by the means of an Optima 7000 DV system from Perkin Elmer. Optical emission from the plasma was viewed axially at a wavelength of 243 nm. Samples were diluted 1:20 in Milli-Q water and assessed in triplicate. Gold concentrations were calculated from a standard curve (2–2000 μg/L), which was established using a gold standard for ICP (38168; Fluka). To counter matrix effects, matching PBS cell culture controls were subtracted from each sample.

### Statistics

To investigate the significance (*p* < 0.05) of the translocation assay, the SigmaStat program for Windows (version 3.10; SYSTAT Software, Inc.) was used. With one-way analysis of variance, pairwise multiple comparison procedure (Student–Newman–Keuls) was tested. Results are presented as mean (*n* = 3) ± standard error of the mean. GraphPad Prism was used to investigate the significance of AuNP translocation data (GraphPad Software, Inc.). Data are represented as mean ± standard deviation (SD).

## Results and Discussion

### Design of the SIMPLI-well

SIMPLIs were conceived with the aim of making the use of ceramic membrane array chips, intended for cell culture of epithelial tissue barrier models, simple and reproducible. This resulted in an insert that fits a six-multiwell plate (Fig. 1A) and is compatible with routine laboratory handling. The system is based on the use of a clamping mechanism, consisting of two cylinders, micromachined from a PC tube, which slide into each other via a bayonet turn-lock movement^20^ (see expanded schematic view of the system in Fig. 1). To lessen the wear generated by the bayonet movement, a thin Teflon O-ring is placed between the chip and the outer cylinder. A silicon O-ring is placed in a groove inside the inner cylinder. This O-ring ensures that any transport between the apical and basolateral compartments is confined exclusively to the microporous membrane array. The novel insert concept is described in more detail elsewhere^20^; it is, however, the first time that the system was assembling the membrane in a plastic holder fitting a standard well plate, which makes it more interesting for many applications. The two cylinders were produced in PC and found to be compatible with multiple autoclave cycles for sterilization purposes and could be reused several times after the cleaning procedure. Upon hanging the system on the well wall, there is a distance of 1.5 mm between the permeable ceramic membranes and the bottom of the well. In this configuration, the tight clamping provides a two-compartment cell growth system while also suspending the ceramic support at the correct distance for standard inverted microscopic observation during culture. The ceramic windows are transparent with no autofluorescence. The square ceramic chips (14 × 14 mm^2^) hold an array of 23 pyramidal microwells with square openings of 1.5 × 1.5 mm^2^, a depth of 380.5 μm, and, at the bottom, a porous surface area of 1.0 mm^2^ as freestanding ceramic membrane. Consequently, each chip presents 23 mm^2^ of porous surface for cell growth, with periodically (hexagonal grid) distributed 1.0-μm holes and 500-nm-high cylindrical walls. Upon system assembling, the overall surface available for the cell growth is roughly 0.8 cm^2^. This makes the size of the support comparable to a commercial 12-well plate insert.

Epithelial cells (A549) were seeded on the SIMPLI-well and grown for 5–7 days. Conventional fluorescence images, after fixing and staining the F-actin cytoskeleton, show homogenous growth of the epithelial cells in monolayers on the silicon nitride membrane, as well as along the silicon slope defined by the pyramidal well area (Fig. 1B). Phase-contrast images of epithelial, as well as endothelial, cells grown on either the SIMPLI support or the PET membranes (3-μm pores) showed that both cell types were able to grow to confluence on either membrane (Fig. 1C).

A number of manufacturers produce porous microwell inserts for cell cultures, including Merck Millipore (Millicell^®^), Thermo Scientific (Nunc™), Corning, Inc. (Transwell™), Greiner Bio-One GmbH (ThinCert™), and BD Biosciences (BD Falcon™). All these are also disposable. The membranes used in these inserts can be divided into two types: polymer membranes and Anapore™ (aluminum oxide) membranes.^27,28^ Polymer membranes made from PET, hydrophilic polytetrafluoroethylene (PTFE), PC, and mixed cellulose esters are available. Pores are introduced by ion track etching, resulting in a random spatial distribution of well-defined pores, described by an average pore density. Typical pore sizes are 0.4, 1, and 3 μm, with pore surface fractions (filling factor) of 0.2–15% and a membrane thickness of 10 μm. Similarly, the Anapore™ membranes can provide uniformly distributed pores and finely tuned pore diameters in the submicron range—however, their thickness:pore diameter ratio is higher and thus disadvantageous with respect to passive particulate diffusion.

The need for robust, thin, biocompatible, and permeable supports, like silicon and silicon nitride, has attracted research efforts from a number of experts in the microfabrication of hard materials. SiMPore, Inc. recently introduced the NanoBarrier™ technology giving excellent results in cell imaging and other applications.^29,30^ Researchers have provided a number of laboratory-scale methods for the preparation of ceramic supports compatible with cell cultures, mostly via their embedding in microfluidic devices.^31^ Additionally, these solutions are compatible with SEM and TEM techniques. Given the physicochemical features of an ultrathin ceramic membrane array chip, the innovative SIMPLI-well system offers all these advantages on a macroscopic area, equal to 23 mm^2^ of permeable surface over 0.8 cm^2^ of surface available for cell growth, where handling procedures are identical to those required for standard commercially available inserts. In addition, the combination of silicon's excellent robustness with the elastic properties of a noncrystalline structure, silicon nitride, as well as the potential to reuse it after cleaning, that is, by wet cleaning using highly oxidizing etchant or autoclaving, are two substantial improvements.

### Characterization of epithelial–endothelial cocultures

Cocultures of epithelial and endothelial cells grown on the new silicon nitride permeable supports were optimized and compared to cultures grown on conventional PET membranes with pore sizes of 1 and 3 μm.

**FIG. 2.**
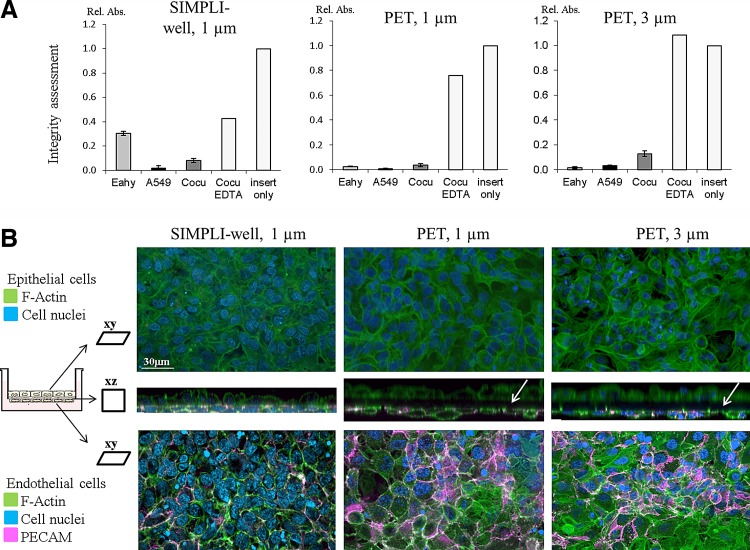
Integrity assessment, cell characterization, and growth of the epithelial and endothelial bilayers. **(A)** As shown by the relative absorbance (Rel. Abs.) at 600 nm, the passage of dextran blue in the endothelial (Eahy)–epithelial (A549) cocultures (cocu) was higher for all supports than for the monolayers but still significantly lower than the positive controls, that is, cultures treated with EDTA or the inserts only. Data are expressed as mean ± standard deviation (SD), *n* = 3 (except for inserts only and EDTA controls, which were only performed once). **(B)** Laser scanning micrographs of bilayers stained for F-actin (green), cell nuclei (blue), and platelet endothelial cell adhesion molecule (PECAM; pink). For each membrane type, a complete z-stack from both sides of the membrane is presented; therefore, the upper images are more blurred since the imaging started at the endothelial cell level. The xz projection (middle image) shows the close cell–cell interactions for the cocultures grown on the SIMPLI-wells compared to both PET membrane inserts, where a black gap between the two cell layers can be seen (white arrows). The xy projections revealed a dense and confluent monolayer of both cell types on the upper and lower sides of the membranes. The endothelial cells expressed the specific endothelial marker PECAM (Fig. 2B, pink).

The dextran blue assay was used to assess the cell layer integrity, that is, the less translocation the tighter the cell layer. Figure 2 shows that the EA.hy926 endothelial cell monocultures were not as tight when grown on the SIMPLI-well compared to those grown on conventional membranes, whereas for the A549 monocultures grown on the three supports, no differences were found (Fig. 2A). Interestingly, the passage of dextran blue through the endothelial–epithelial cocultures was higher for all supports than for the monolayers but was still significantly lower than the positive controls, that is, cultures treated with EDTA or the inserts only. The EDTA control for the SIMPLI-well was less effective in comparison to the two commercial PET membranes, indicating a much stronger cell–cell interaction. We have, however, tested a longer EDTA incubation time (several hours), which also resulted in 100% dextran blue translocation (data not shown).

Regardless of the *in vitro* model used in transport or translocation studies, the first priority is always to ascertain the integrity of the model.^5,32^ The optical density of dextran blue in the lower chamber in all cocultures on the various supports was more than an order of magnitude lower than values measured beneath a membrane without cells, similar to other studies,^33^ indicating a functional epithelial–endothelial barrier. It is important to mention that the cocultures show a higher permeability of the tracer dye compared to the epithelial monocultures, which is in line with observations made by us among others,^13,34^ and indicate that the cells interact with each other either directly or by secretion of soluble factors. Tight epithelial–endothelial bilayers, observed by LSM, support the functional barrier integrity.

The cell morphology and expression of specific endothelial markers were investigated by LSM (Fig. 2B). The A549 epithelial cells and the EA.hy926 endothelial cells grown on the upper and lower sides, respectively, of all different supports showed a confluent growth with a monolayer appearance. The epithelial cells, shown on the upper side, appear blurry since the endothelial cells were closer to the objective, with a membrane between. The xz sections (middle images) show close cell–cell interactions for the cocultures grown on the SIMPLI-wells in contrast to the black gap found between cells cultured on PET membranes. Endothelial cells were identified by the expression of a PECAM marker (Fig. 2B) and the vWF (Supplementary Fig. S2), neither of which was detected in epithelial cells. In addition, expression of E-cadherin was shown in epithelial cells (Supplementary Fig. S2); however, since also a weak staining was seen in endothelial cells, this marker was not used for further experiments.

### Quadruple cocultures

**FIG. 3.**
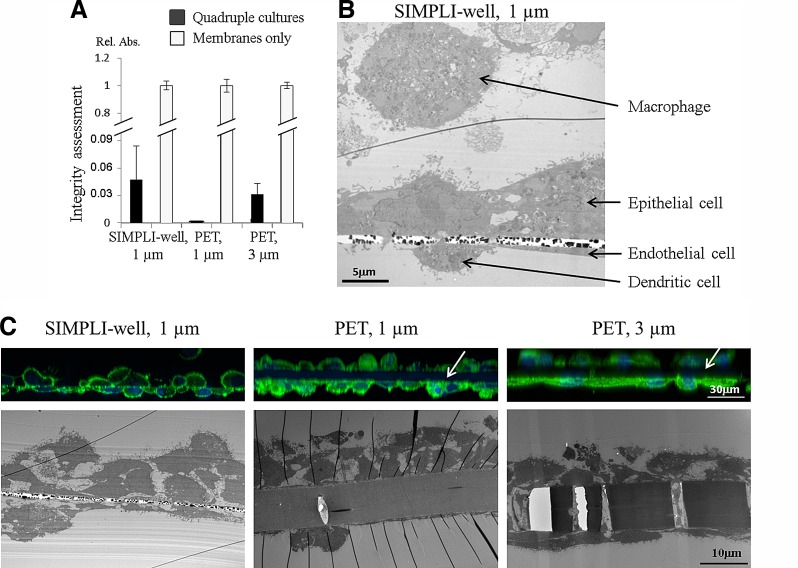
Characterization of the quadruple cocultures. Quadruple cultures composed of epithelial–endothelial bilayers supplemented with monocyte-derived dendritic cells on the endothelial side and monocyte-derived macrophages on the epithelial side. **(A)** As shown by the dextran blue assay, epithelial–endothelial integrity remains intact after addition of immune cells. Compared to the inserts only (white bars), quadruple cultures allow only little dextran blue to pass through (black bars). Data are expressed as mean ± SD, *n* = 3. **(B)** The quadruple cocultures grown on the SIMPLI-well were fixed and prepared for transmission electron microscopy (TEM), showing a confluent epithelial and endothelial layer on each side of the supports, in addition to the respective immune cells on both sides. **(C)** Comparison of the quadruple cocultures grown on the different supports. The upper images represent laser scanning micrographs of bilayers stained for F-actin (green) and the cell nuclei (blue). For each membrane type, an xz projection from a complete z-stack from both sides of the membrane is presented; therefore, the upper images are more blurred since the imaging started at the endothelial cell level. The white arrows point to the black gap between the two cell layers for the two PET membranes. The lower images show TEM micrographs. Note the thickness of ∼10 μm of both PET membranes in comparison to the 0.5-μm-thin porous silicon nitride support.

The quadruple cocultures, composed of epithelial–endothelial bilayers supplemented with MDDCs on the endothelial side and MDMs on the epithelial side, were prepared. Epithelial–endothelial integrity persists after the addition of immune cells to the coculture (Fig. 3A). TEM shows a confluent epithelial and endothelial layer on each side of the support, in addition to the respective immune cells on both sides (Fig. 3B). The quadruple cocultures were also grown on the different supports for comparison. The cell morphology is similar for all three conditions; however, the contrasting thickness of both PET membranes (∼10 μm) in comparison to the thin silicon nitride porous support (Fig. 3C) is obvious.

Regarding surface expression, A549 cells express the epithelial-specific protein E-cadherin, and the two immune cells express their specific surface receptors, such as CD14 (MDM) and CD86 or CD83 (MDDC)^11^ (data not shown). The EA.hy926 cells, used for the first time in these co- and quadruple cultures, were investigated with respect to specific endothelial characteristics, such as the expression of vWF^35^ and PECAM-1,^36^ and both endothelial-specific proteins were detected in the endothelial cells (data not shown).

### Translocation of AuNPs across the quadruple cultures grown on different supports

**FIG. 4.**
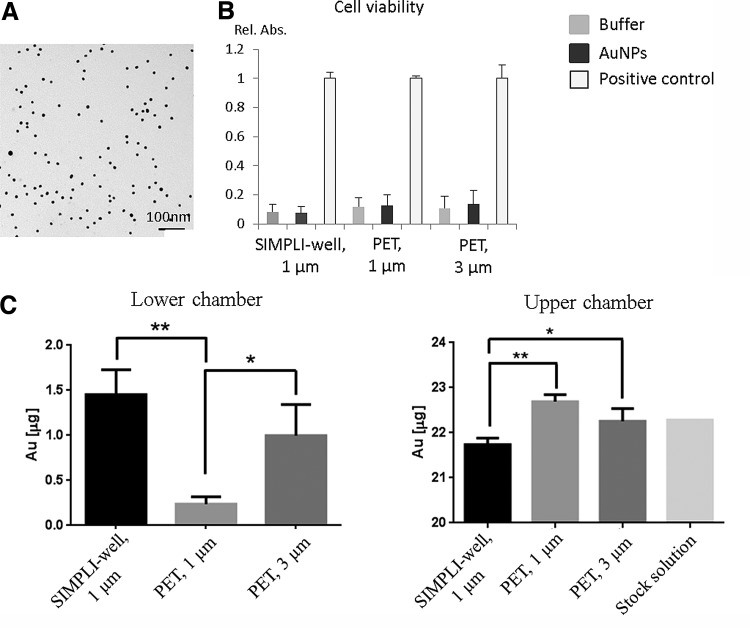
Translocation of gold nanoparticles (AuNPs) across the quadruple cocultures grown on different supports. **(A)** TEM image of polyvinyl alcohol (PVA)-functionalized AuNPs. Note the PVA coating is not visible by TEM. **(B)** Exposure of the quadruple cultures to AuNPs did not induce cytotoxicity as measured by lactate dehydrogenase (LDH) release. Cells exposed to the buffer only were used as negative controls, and Triton X-100 was used as the positive control for the cytotoxicity assay. **(C)** The Au content in the lower chamber, measured by inductively coupled plasma optical emission spectroscopy (ICP-OES) after 2 h in quadruple cultures grown on the SIMPLI-wells, was slightly higher than in the case of cultures grown on conventional PET membranes bearing 3-μm pores, whereas significantly less Au was detected for cells grown on the conventional PET membranes with 1-μm pore size. Data are expressed as mean ± SD, *n* = 3 (except for the stock solution, which was only performed once). *p < 0.05; **p < 0.01.

One family of nanomaterials that has attracted a lot of interest concerning biological applications is that of gold.^37^ AuNPs are readily incorporated by many different types of cells and have been found to be suitable for use in nanomedicine since they show low toxicity.^38,39^ We have used PVA-coated AuNPs with a hydrodynamic diameter of 42.2 nm (Fig. 4A, Supplementary Fig. S1) and a zeta potential of −13 mV (in PBS) to compare their translocation behavior in the quadruple cocultures grown on the different supports. The premixed AuNP suspension (22.3 μg/mL, 1 mL in total) was added to the top of each insert, and the Au content in the medium of the upper and lower chambers was determined by ICP-OES after 2-h suspension exposure. This exposure did not impair the membrane integrity as determined via the dextran blue assay (data not shown). In addition, no cytotoxicity (Fig. 4B) was observed in the presence of AuNPs relative to untreated controls. The Au content in the lower chamber after 2 h in quadruple cocultures grown on SIMPLI-wells bearing 1-μm pores was slightly higher than in the case of cultures grown on PET membranes bearing 3-μm pores, whereas significantly less Au content was detected for cells grown on the PET membranes with a 1-μm pore size in comparison to the SIMPLI-wells (Fig. 4C). The efficient translocation of Au across the cultures on the silicon nitride porous supports was also reflected by the fact that the lowest Au content was found in the upper chambers (Fig. 4C).

The majority of the Au was detected in the upper chamber after 2 h. While about 1% was translocated in the quadruple cell model grown on PET membranes with 1-μm pores, about 4.5% was translocated using the conventional membranes with 3-μm pores and 7% for the silicon nitride porous supports with 1-μm pores. A comparison of these translocation rates with any human data is currently not possible, while only rates for mice or rats could be found for different Au nanoparticle sizes, concentrations, and time points. These translocation fraction values range from 0.2% to 8%^40–42^ and are in line with our observations, although different particles in terms of size and polymer coatings have been used, and further experiments will be needed in a more coordinated approach. In addition, a comparison and/or correlation between different species is still lacking.

## Conclusions

A host of sophisticated 3D models of the air–blood tissue barrier have been recently developed, including complex cocultures^11–13^ and microfluidic systems mimicking the breathing and diseased lung.^9,10^ However, all these models fail to mimic one important anatomical feature of the air–blood tissue barrier in humans: its submicron thinness.^15,16^ This parameter is essential for accurately modeling the interactions between different cells in the barrier, as well as for the translocation behavior of any material, which is deposited on the apical lung cell surface.

All epithelial coculture systems neglect the fact that the cells have to be grown on thick, polymer-based cell culture inserts, which do not mimic the structure and function of the basement membrane. A new solution is provided here to overcome this issue by the design of a new ultrathin ceramic membrane and thereby improving a coculture model of the air–blood tissue barrier. The new quadruple system has been fully characterized, revealing the presence of cell type-specific differentiation markers as well as the optimal spatial arrangement of the cells. In future studies, it might also be interesting to include primary (lung) cells or to adapt the fabrication of the ceramic inserts for microfluidic devices mimicking an optimal physiological environment through the inclusion of flow.

We are currently, to the best of our knowledge, the first team worldwide that provides an innovative new support for any biomimetic epithelial tissue model with the proof of concept for an optimized lung tissue. This approach offers a unique opportunity to obtain a fundamental understanding of the complex processes, that is, the kinetics of drugs or NPs, occurring at any biological barrier in humans.

## Supplementary Material

Supplemental data

Supplemental data

## Abbreviations Used

AuNPs: gold nanoparticles
BSA: bovine serum albumin
DDLS: depolarized dynamic light scattering
FBS: fetal bovine serum
ICP-OES: inductively coupled plasma optical emission spectroscopy
LDH: lactate dehydrogenase
LSM: laser scanning microscopy
MDDCs: monocyte-derived dendritic cells
MDMs: Monocyte-derived macrophages
PALS: phase amplitude light scattering
PBS: phosphate-buffered saline
PC: polycarbonate
PECAM-1: platelet endothelial cell adhesion molecule-1
PES: polyethersulfone
PET: polyethylene terephthalate
PFA: paraformaldehyde
PTFE: polytetrafluoroethylene
PVA: polyvinyl alcohol
SC-1: standard clean 1
SD: standard deviation
SEM: scanning electron microscopy
SIMPLI: Silicon nitride Microporous Permeable Insert
TEM: transmission electron microscopy
vWF: Von Willebrand factor
