# Supplementary material for: Ultrathin Ceramic Membranes as Scaffolds for Functional Cell Coculture Models on a Biomimetic Scale
Source: Biores Open Access. 2015 Dec 1;4(1):457–68. doi: 10.1089/biores.2015.0037 (PMC4691652; doi:10.1089/biores.2015.0037)
Supplement: Supplemental data [file Supp_Figure1.pdf]

Supplementary Data

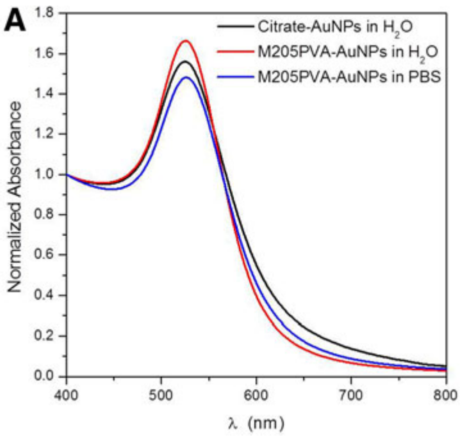

B

Hetero-functionalized Au NPs	Hydrodynamic diameter [nm] (Polydispersity)*+	Zeta Potential [mV] (SD) H2O	Zeta Potential [mV] (SD) PBS Ph7.4
Citrate	20.2(30%)	-32(2)	aggregated
Thiolated PVA (M205)	42.2(31.5%)	-5(3)	-13(5)

SUPPLEMENTARY FIG. S1. **(A)** UV–Vis spectra of citrate gold nanoparticles (AuNPs) and polyvinyl alcohol (PVA)–AuNPs in H_2O and phosphate-buffered saline (PBS). **(B)** Hydrodynamic diameter obtained by dynamic light scattering (DLS) and zeta potential of citrate- and polymer-coated AuNPs. *+Polydispersity (%).
