# Supplementary material for: Ultrathin Ceramic Membranes as Scaffolds for Functional Cell Coculture Models on a Biomimetic Scale
Source: Biores Open Access. 2015 Dec 1;4(1):457–68. doi: 10.1089/biores.2015.0037 (PMC4691652; doi:10.1089/biores.2015.0037)
Supplement: Supplemental data [file Supp_Figure2.pdf]

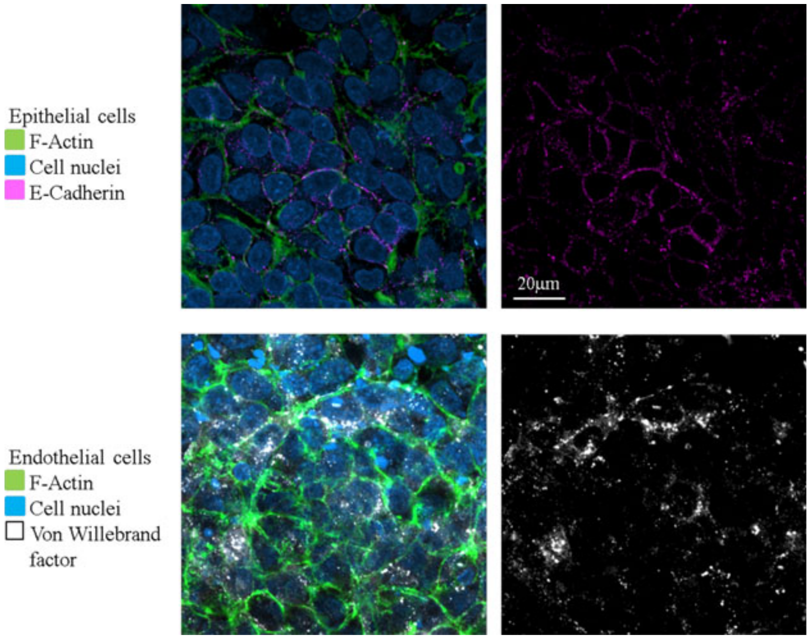

SUPPLEMENTARY FIG. S2. Laser scanning micrographs of epithelial cells (A549) stained for F-actin (green), cell nuclei (blue), and E-cadherin (pink) and endothelial cells (Eahy) stained for F-actin (green), cell nuclei (blue), and Von Willebrand factor (white). The images represent single xy layers.
